# Towards a Modular On-Premise Approach for Data Sharing

**DOI:** 10.3390/s21175805

**Published:** 2021-08-28

**Authors:** João S. Resende, Luís Magalhães, André Brandão, Rolando Martins, Luís Antunes

**Affiliations:** 1 Faculty of Sciences, University of Porto, 4169-007 Porto, Portugal; up201606761@fc.up.pt (L.M.); andre.brandao@fc.up.pt (A.B.); rmartins@dcc.fc.up.pt (R.M.); lfa@fc.up.pt (L.A.); 2 Institute for Systems and Computer Engineering, Technology and Science (INESC-TEC), R. Dr. Roberto Frias, 4200-465 Porto, Portugal

**Keywords:** cloud-of-clouds, multi-party computation, data sharing, privacy, machine learning

## Abstract

The growing demand for everyday data insights drives the pursuit of more sophisticated infrastructures and artificial intelligence algorithms. When combined with the growing number of interconnected devices, this originates concerns about scalability and privacy. The main problem is that devices can detect the environment and generate large volumes of possibly identifiable data. Public cloud-based technologies have been proposed as a solution, due to their high availability and low entry costs. However, there are growing concerns regarding data privacy, especially with the introduction of the new General Data Protection Regulation, due to the inherent lack of control caused by using off-premise computational resources on which public cloud belongs. Users have no control over the data uploaded to such services as the cloud, which increases the uncontrolled distribution of information to third parties. This work aims to provide a modular approach that uses cloud-of-clouds to store persistent data and reduce upfront costs while allowing information to remain private and under users’ control. In addition to storage, this work also extends focus on usability modules that enable data sharing. Any user can securely share and analyze/compute the uploaded data using private computing without revealing private data. This private computation can be training machine learning (ML) models. To achieve this, we use a combination of state-of-the-art technologies, such as MultiParty Computation (MPC) and K-anonymization to produce a complete system with intrinsic privacy properties.

## 1. Introduction

In recent years, companies have adopted a growing trend to migrate their services, data, and applications to cloud-based infrastructures, often attracted by reduced costs, availability, and flexibility. With the cloud, companies have the flexibility to perform dynamic workloads without ever needing additional hardware, allowing them to store new types of data or create new business opportunities without significant infrastructure commitments. However, the migration to the cloud is a process that involves solving new security and privacy issues that were non-existent for on-premise deployments. In the cloud, the lack of visibility and transparency about data processing leads to users losing control over their data [1]. Companies need to analyze the cost of having their privacy compromised due to some security issues regarding cloud migration that are not solved yet [2].

Over the years, many technologies have emerged [3] to reduce users’ data exposure to unauthorized parties, namely, anonymization tools and cloud-of-clouds. Data analysis without knowledge of plain text data is one of the most promising challenges for ensuring user privacy. Data privacy has been a growing concern over the years. For example, Android and iOS apps collect users’ data, namely their behavior, and companies benefit from these data. It is important to note that, even if companies do not collect behavioral data, much of the data sent by mobile devices may be considered sensitive or identifiable [4], such as IPs, GPS, and MAC addresses, requiring processing to ensure that the collected data are no longer able to identify an individual.

The issue with re-identification—the process of identifying an individual using the collected data—is even greater in cases where data collected by different companies are aggregated, creating a larger dataset and increasing the likelihood of re-identification [5].

Due to these facts, it is of utmost importance to create pseudo-anonymization protocols so that it is possible to collect patterns about the population without uniquely identifying a user. To this end, this paper explores the usage of anonymization techniques, such as the removal of HIPAA. The HIPAA identifiers are managed by a HIPAA Privacy Rule that establishes national standards to protect individuals’ medical records and other personal health information and applies to health plans, such as name, phone number, address, or postal code. The identifiers, provided by the HIPAA privacy rule, sets forth policies to protect all individually identifiable health information that is held or transmitted [6]. We also explore the use of MPC (reviewed in Section 3.4) to exchange information without sharing the cleartext dataset with the other party. MPC provides the critical notion of learning with encrypted data, increasing the trust level and strengthening privacy properties.

In this work, we present a solution that improves user privacy based on a hybrid architecture composed by a local server capable of performing anonymity, MPC, and encryption as well as a public cloud storage component, based on the cloud-of-clouds concept, which is responsible for storing the data received from users confidentially and integrally, providing an extra layer of security with the *data at rest*. *Data at rest* in information technology means data that are housed physically on computer data storage in any digital form [7].

Our solution covers the following goals:

- Maintains data privacy on the public cloud storage;
- Enables cooperation between institutions, through data sharing, without compromising privacy;
- Enables data sharing among users;
- Uses secure enclaves to increase trust on the private key management;
- Reduces, as much as possible, the performance overheads.

The paper is organized as follows: Section 1 is the introduction and contextualization where we define the assumptions and main contributions. Section 2 contains the related cloud storage systems and background knowledge about federated learning. In Section 3, we detail the architecture and the specific implementations/features available in the system. Section 4 includes the security evaluation of the system, and Section 5 contains tests performed on the platform, focusing first on the enclaves technologies and then on the system scalability. Lastly, Section 6 is the future work direction and the conclusion of the work.

### 1.1. Assumptions

This work assumes that the on-premise server is trustworthy, and therefore, this server is capable of receiving cleartext data from users, performing all privacy-preserving operations, and storing the data off-premise. The threat model assumed for the on-premise server is the typical threat model applied to Intel SGX enclaves, specifically in a trusted cloud environment [8,9]. SGX allows the operating system and user-level code to define private regions of memory, called enclaves, whose contents are protected and cannot be read or saved by any process outside the enclave itself, including processes running at higher privilege levels. Additionally, for the system, we assume that any internal process, such as sync information, can be performed using master–slave approaches.

### 1.2. Contributions

The work presented here provides the following contributions:

- Run-time Adjustable Privacy Schemes (RAPS): Given the multitude of possible workloads for which our solution aims to provide support for data sharing, MPC, cloud-of-clouds, we have designed an adjustable privacy mechanism that enables us to tweak the anonymization, storage location, and persistence parameters, allowing them to have more control over the processing that their data might suffer.
- Anonymization of sensitive data: When the system detects, using the parameters established on the RAPS, a possible privacy leakage or any other identifiable attribute defined on the scheme, it stores in on-premise storage that specific attribute identified from the dataset. This step is essential before sharing information with other parties, as it enhances anonymization and privacy.
- Secure sharing with Machine Learning: When we analyze the data-sharing mechanism on the cloud, we usually have one option to share the entire content of the file. We extend this feature with the possibility of sharing information after removing personal identifiers to allow learning, using MPC or HE protocols.
- Cloud-of-clouds deployment: The cloud-of-clouds deployment ensures that the *data at rest* are not entirely accessible to any public cloud provider. The different clouds only have the vision of a part of the encrypted data. Even if they uncover the encryption keys, they can only access a portion of the file.
- SGX integration: We use the Public Key Infrastructure (PKI) as the basis of secure client–server communication, which is based on *HTTPS*. However, issues related to the private key storage protection on a PKI infrastructure force us to adopt hardware-based solutions. With Intel SGX, we prevent the exposure of the private key by attackers since it never leaves the enclave. This private key protection layer ensures that, even if the system is compromised, the private key associated with the *HTTPS* communication is secure on the enclave and inaccessible to the attacker, preventing the disclosure of previous *HTTPS* communications.
- Implementation Prototype: We have a working implementation prototype described in this paper. The server includes all the cryptographic computations, being those built on top of *OpenSSL* and the SGX infrastructure. Our implementation focuses on confidentiality, integrity, availability, persistency, and privacy preservation. In addition to the on-premise server, our implementation contains a front-end Android app capable of fully interacting with the on-premise services.

## 2. Background and Related work

This section presents several state-of-the-art technologies and techniques that provide some of the aspects required for the architecture, namely cloud storage, anonymization, and cooperation through private ML training.

### 2.1. Cloud Storage

Cloud computing is a distributed computing paradigm that focuses on providing users with distributed access to scalable, virtualized hardware and software infrastructure over the Internet [10]. There are multiple types of cloud, namely, public, private, and hybrid clouds [10]. The most used cloud types are the public cloud-based storage services, which are an inexpensive and scalable way for companies and users to create and maintain their files, with high availability and redundancy against data loss [11].

These resources can be rapidly configured and deployed with minimal management efforts. The cloud acts as a data storage device placed on the internet, free or paid, accessible from any platform. This type of technology has several advantages, namely: availability on any platform or device; cost savings (users only need an internet connection remotely, without needing to buy hard drives with large storage capacities); and ease of use in making backups and restoring the information [12,13]. The lack of local data maintenance and the absence of local storage hardware are the main advantages of this type of storage. Its availability is driving the adoption of this type of storage.

However, this accessibility to users raises and challenges privacy and security concerns. It focuses on the emergent regulations that create challenges for data management and user awareness [3]. For cloud solutions, multiple protocols can enhance the user’s privacy and security regarding data encryption communication (Section 3.3), but cloud providers’ trust is also limited to the security and privacy of the provider. A specific extensive comparison between the different types of cloud providers is described by *Sumit Goyal* [10].

**Figure 1:**
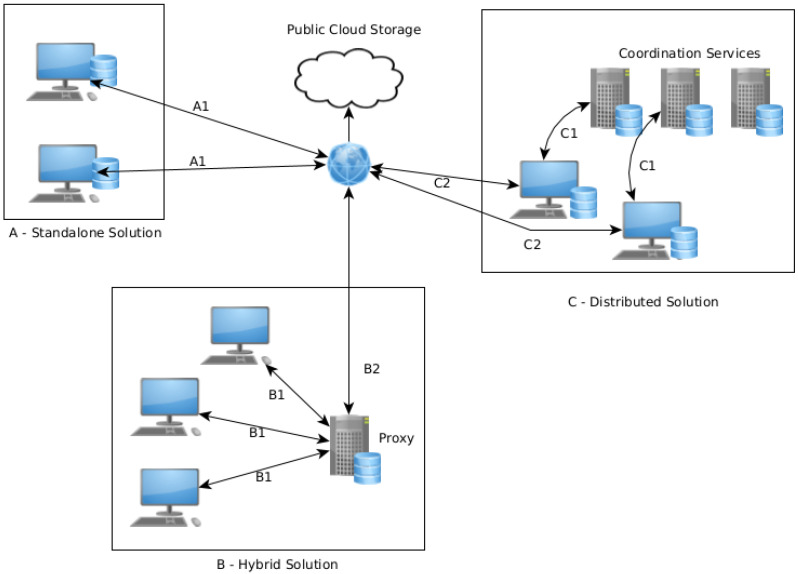
Different cloud systems.

Figure 1 demonstrates the three main types of data manipulation: standalone, hybrid, and distributed. The following subsection will overview each of them, identify advantages and disadvantages, and overview the existing implementations.

The following systems can use a single public, private cloud provider or multiple secure storage systems to take advantage of cloud computing benefits by combining multiple clouds. Using multiple clouds is called cloud-of-clouds. In this scenario, the user can encrypt and send the information to multiple providers rather than relying on one cloud provider’s security.

#### 2.1.1. Standalone

In this model, the user retains control over the data, but the user devices assume the responsibility of performing all the encryption and coordination mechanisms to manage the information locally or in the cloud providers. Figure 1 shows that there is only a direct connection (represented by the connection B1) between the cloud and the user. Table 1 shows the advantages and disadvantages of standalone cloud storage.

The systems that represent this type of mechanism are as follows:

- *S3FS* [14]: a filesystem in userspace (FUSE) that connects directly with buckets from Amazon that allow the encryption of data on the Amazon infrastructure;
- *S3QL* [15]: similar to *S3FS* with support to all types of files, such as symbolic links, *S3QL* can detect duplicated files and reduce the amount of space required to store it;
- *rClone* [16]: in contrast to the previous system, *rClone* only focuses on uploading/downloading the content of the folder. It can be considered a synchronization tool between folders of the filesystem.

#### 2.1.2. Hybrid

The *hybrid* mechanism uses a proximity server, such as an edge component, in the network to increase the upload and download speed, compared to a public cloud provider.

Figure 1 represents the *hybrid* cloud system in the A rectangle, and there are two main connections: the B1 (from the client to the *hybrid* solutions) and B2 (from the *hybrid* solutions to the public cloud). Table 2 shows the advantages and disadvantages of hybrid cloud storage.

*BlueSky* [17] focuses on offloading computing to a simple cloud provider instead of splitting the information over multiple public cloud providers. However, unlike the *standalone* version, this solution makes the security model rely on a relay server, which means that the information is in cleartext on the *BlueSky* server and is only encrypted when uploaded to the cloud. Despite having a single point of failure, this type of solution allows the system to become more usable, as the encryption process can be performed on the server. Argus [18] is a similar approach but focus on the protection over multiple cloud providers.

#### 2.1.3. Distributed

The *distributed* version removes the single point of failure of the *hybrid* approaches. The user assumes responsibility for sharing the information with the cloud provider, but uses a set of coordination servers to decide where to store the information in the cloud, allowing functionality to be maintained, such as sharing files between different users. Figure 1 represents the *distributed* cloud system (rectangle C, where C1 represents the connection with the coordination service to store the files in the cloud using C2). Contrarily to the *hybrid* approach, it is always the client who performs the upload to the cloud on the *distributed* version.

These solutions ensure that the user never loses access to the information, ensuring regular backups to maintain the data, contrarily to the *standalone* solutions, which require manual intervention. So, the *distributed* approach is better regarding user protection against attacks, compared with the *standalone* version. This type of solution offers the same number of features as the *hybrid* approach, but when compared to the *standalone*, it provides more features, as it is possible to encrypt the data locally and share. Table 3 shows the advantages and disadvantages of distributed cloud storage.

*Distributed* solutions are an active line of research in the field of cloud storage. Multiple solutions take this approach, namely *Depsky* [19] which is a storage cloud-of-clouds. It overcomes the limitations of individual clouds by using cloud-of-clouds in which the operations (read, write, etc.) are implemented using a set of Byzantine quorum systems protocols. *SCFS* [20] is based on *Depsky*, but it relies on FUSE to allow users to interact with the filesystem stored directly in the cloud. More recent solutions, such as *RockFS* [21], have client-side protection against specific attacks, namely protection against ransomware, protection of the credential stored locally, or attacker access files on the local file system. Lastly, *Charon* [22] from the same research group of *Depsky* and *SCFS*, has support for big data processing and usage of *BFT* for coordination, such as the remaining solutions. All of these solutions can be considered an evolution of the previous solutions. Our main focus is to enhance these concepts with privacy-preserving techniques, focusing on data and information management throughout the cloud.

### 2.2. Privacy Preserving

Privacy is one of the essential properties of machine learning. In this section, we briefly review and compare different privacy techniques that can be used for protecting federated learning [23,24,25].

#### 2.2.1. Multiparty Computation

MPC is a subfield of cryptography that involves communications between multiple parties, providing security proofs in a well-defined simulation framework to ensure complete zero-knowledge, i.e., each party knows nothing but its input and output. Zero-knowledge is highly desirable, but this property often requires expensive computing protocols that compromise protocol efficiency. Sometimes, partial knowledge disclosure can be acceptable if security guarantees are provided (for example, removing personally identifiable information). Recently, SecureML [26] used the MPC concept to train machine learning models with two servers and semi-honest assumptions. MPC protocols are used for model training and verification without users revealing sensitive data to each other and the network (data never leave the infrastructure).

#### 2.2.2. Differential Privacy

Differential privacy [27] can also be used for data protection. The methods of differential privacy involve adding noise to the data or using generalization methods to obscure certain sensitive attributes until the third party cannot distinguish the individual (an example of this is shown by Ricardo Mendes et al. [28]). However, the methods still require data to be transmitted from the device to an unknown external server, which often involves a trade-off between accuracy and privacy.

#### 2.2.3. Homomorphic Encryption

Homomorphic encryption [29] is also used to protect user data during machine learning training. Unlike differential privacy, the data and the model itself are not transmitted elsewhere, nor can they be disclosed to the other party. Therefore, there is a small possibility of leakage at the raw data level. Recent work has adopted homomorphic cryptography to centralize and train data in the cloud [30,31]. Many times, MPC and homomorphic encryption work together to attain the best performance when performing ML tasks. An example of this is the library Secureml, which relies on MPC for many tasks but for some specific process, it uses HE; for example, for the offline protocol, it uses linearly homomorphic encryption [26].

## 3. Architecture Overview

**Figure 2:**
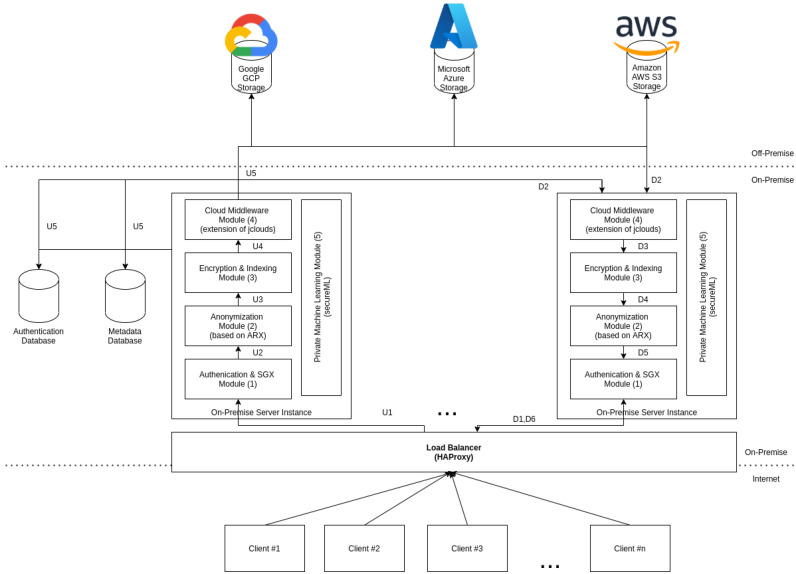
Architecture overview.

The overall system architecture, represented by Figure 2, contains three main components: clients, cloud providers, and the on-premise server.

An Android device represents the client with an application capable of sending information to the on-premise server. The on-premise server is the most critical component, as it performs all the necessary tasks to ensure that the clients can use public/private cloud storage. The on-premise server is composed of five main modules:

- Authentication and SGX module: it is capable of performing user authentication and serves as TLS termination for the remaining of the on-premise server. The private key associated with the TLS communication is stored on the secure enclave, it being the responsibility of this module to manage the SGX secure enclave as well.
- Anonymization module: it enables the anonymization of datasets, documents, photos, or videos, according to the currently active RAPS. This module is also responsible for creating and managing RAPS.
- Encryption and indexing module: it is capable of leveraging algorithms that ensure confidentiality and integrity on the data to be uploaded to the cloud. All the metadata produced by this process is stored on an on-premise database.
- Private machine learning module: it is based on *SecureML*, enabling two distinct organizations to train an ML model using their private information, but neither organization has access to the raw private information, only encrypted versions of it, improving privacy and mitigating uncontrolled distribution of private data.
- Cloud middleware module: it is based on jClouds, and ensures the proper persistence of the data uploaded across the cloud-of-clouds by using erasure encoding techniques. The component is also responsible for erasure by decoding the data on a download request and providing access to the private cloud storage.

Our architecture is designed to be easily scalable by reducing the number of states that the on-premise server manages locally, which allows the on-premise server to be easily replicated when required. Placing a load balancer between the users and the on-premise server allows loading balance of the requests across all the replicas instantiated on-premise, allowing the system to scale with the increasing number of concurrent users.

In our architecture, the user needs to be authenticated with the on-premise server to upload a file and communicating via *HTTPS*; otherwise, the connection is refused by the Authentication and SGX module (1). When the user uploads a file, Figure 2 is left, the content is analyzed according to the file type and active RAPS. If personal data are detected, it splits the information according to the anonymization algorithm that applies to the received file type. Depending on the file type, automatic personal data detection may not detect all the personal data on the file. To cope with this, we allow the user to send the file together with a list of portions of the file content that contain personal information. The anonymization uses that information to redact the personal data from the received file, storing the private information on the private cloud (2). After an anonymization process, two files are generated: one stored on the private cloud, containing the personal data, and the other stored on the public cloud, containing the remaining data. Data must be encrypted and split (3) before uploading it to the different public cloud providers configured (4). A hash is generated for the split data chunks used to verify the integrity of the data on the download process. The hash, the encryption keys, and additional file metadata are stored in an index, allowing the on-premise server to know which files are stored and how to retrieve them. The upload process can be seen as a sequential process that starts in U1 up to the storage in U5. Contrarily to the upload of a file, the download is more complicated. It consists of the same processes, but the order is different. First, the user is authenticated in the system (D1), then the information is collected from the cloud-of-clouds and private storage (D2), then the file recovers the original format and is checked against the HMAC (D3), and finally, the final step is the analysis of the personal identifiers (D4) to recreate the final file to return to the client (D5).

In addition to the process of uploading and download files, we also allow on-premise server instances on different organizations to jointly compute ML models, using privacy-preserving multi-party computation protocols (5).

In the following sections, we will discuss each module in more detail, specifying the implementation details.

### 3.1. Authentication and SGX Module

Internet transactions create security challenges, such as impersonation attacks, in which the attacker can present her/himself as a trusted entity. Franco et al. [32] describe an example attack where a fake trusted entity tries to use or extract information from the source to make the user believe that they are talking to a legitimate source.

These types of compromises can then affect authentication systems or allow them to reveal confidential information exchanged in previous transactions. The authentication and the session key’s management are essential for us, as an attacker can compromise the current and previous communications by only accessing the private key or the session key of an *HTTPS* server. This problem increases if the information is sent in cleartext to the on-premise server. In this scenario, the user must trust the server, so it is paramount that the private key is kept private in any way. To do this, we intend to include all the crucial parts of the computations inside the SGX and use an authentication system to delegate the authorization and authentication to manipulate and request files from the cloud. With systems like SGX, even privileged code is blocked from reading and altering enclave memory: illegal accesses result in CPU exceptions, and read access to enclave memory will always return 0xFF [8].

Similar systems do not address this issue, as they assume a client that performs uploads to and management of the cloud [22], or that the server is trusted and not vulnerable to impersonation attacks.

In our implementation, we followed the idea from André Brandão et al. [33], where the authors modified the Apache webserver SSL module to integrate with Intel SGX, enabling protection against attacks that attempt to compromise the private key. We used the Apache SSL module from the same authors to create a TLS termination point proxy, where all the communications to the premise must travel through before reaching the remainder of on-premise modules. In this proxy, the authentication verification is performed, denying access to the remainder of on-premise modules if the requests are unauthenticated. For the authentication, we used the KeyCloak [34], which uses tokens to assess the identity of the users behind the request.

### 3.2. Anonymization Module

Data anonymization is a process by which the personal data are altered to not enable identification by any party that has access to the anonymized contents. Data anonymization increases user privacy and maintains data usability for data analysis procedures, including ML training algorithms. Depending on the model, ML algorithms usually do not require identifiable information (information that can directly or indirectly identify an individual) to be present in order for them to provide satisfactory results. These procedures often look to sensitive attributes, such as illness or income that, once successfully de-linked from an individual, can be used without imposing risks of identifying the individual and, therefore, not compromising their privacy.

The anonymization module is connected to the output of the Authentication and SGX module, meaning that the requests it receives are from authenticated users. When the anonymization module receives a file, it first checks the file type and loads the anonymization algorithm that best deals with that kind of file type. In our implementation prototype, we only deal with the CSV file type anonymization, using K-Anonymity. Then, the anonymization module checks whether the user has added some information about personal data regions on the file, and if they have, that information is passed to the anonymization algorithm that anonymizes the file. Otherwise, the anonymization module checks the file for HIPAA identifiers and passes that information to the anonymization algorithm as personal information to be removed. The remainder anonymization parameters are defined on the active RAPS.

In more detail, K-Anonymity, created by L. Sweeney [35], is a formal model of privacy that aims to make each record indistinguishable from a defined *K* number of other records. This way, an attacker cannot identify the individual represented by the record, as there are K−1 other records with similar information.

A dataset is considered K-Anonymized when, for any data record with a given set of identifiable attributes, there are at least K−1 other records with the same attributes. To simplify the anonymization of a dataset, the K-Anonymity assigns properties to data attributes that should be treated differently depending on its risk of identifying the individual. There are three types of attributes: Key, Quasi-identifier, and Sensitive.

Key attributes can identify an individual directly without needing access to any external information, such as names, emails, and social security numbers. Key attributes require removal or obscurity, and the latter can be performed using translation tables.

Quasi-identifiers are attributes that can, with the help of external information, identify an individual. Quasi-identifiers, such as zip-codes, birthday, and gender, are the main concern of K-Anonymity, requiring suppression or generalization so that, at least, there must be K data records with similar quasi-identifiers.

Sensitive attributes are those that an individual is sensitive about revealing, such as income or type of illness, that must be de-linked from the individual to ensure individual anonymity.

The anonymization module is also responsible for managing RAPS. When the RAPS need to be modified, the on-premise server administrator issues new RAPS, and that change has an immediate effect on all the future incoming connections without needing a server restart, enabling us to achieve the run-time adjustability that our solution requires. The active RAPS are stored on a synchronized database to be accessible to all the on-premise server replicas accepting connections. It is implemented in the master–slave approach, where the master is responsible for delegating to the slaves the information.

After the anonymization process, the anonymized data are sent to the encryption and indexing module to process the anonymized file for secure off-premise cloud storage.

### 3.3. Encryption and Indexing Module

The generation and management of encryption keys must be kept as secure as possible, using international standards. To achieve this, we will focus on the requirements of NIST [36] to create a state-of-the-art tool. The indexing process locally encrypts all information about the data uploaded to the cloud, including a hash to detect possible unauthorized changes to the block in the cloud after storage.

In our implementation, for each file uploaded, a new AES 256 bit key is generated, and the file is erasure-encoded using Reed Solomon encoding [37], resulting in a series of file shards. A database shard, or simply a shard, is a horizontal partition of data in a database or search engine. Each shard is held on a separate database server instance to spread load. [38]. These shards can become corrupted without losing any information of the file when it is reconstructed. We set our Reed Solomon encoding to output three shards: two of data and one of parity, meaning that we can lose one shard of data or parity, and the system can reconstruct the file without any data loss. To each of the shards outputted by the Reed Solomon encoding, we generate a random 128 bit IV and encrypt the shard, using the AES-CBC with the key created for the file and the IV generated for the shard. To the encryption result, we perform integrity protection in the form of an HMAC. HMAC allows us to add integrity and authenticity properties to the shard on top of the confidentiality provided by encryption. The key used for HMAC is generated for each new file created.

All the metadata generated by this process, namely AES key, HMAC, and IVs, are stored on the synchronized metadata database (similar to the RAPS database) on a new entry that contains the file name and the user that published the file. The encrypted shards are sent to the cloud middleware module that sends each one of the encrypted shards to a different cloud provider, limiting the sovereignty that each provider has over the data.

### 3.4. Privacy-Preserving Machine Learning Module

The privacy-preserving ML aims to extract useful information from data, while privacy is maintained by concealing information. Thus, it seems difficult to harmonize a system that incorporates these competing interests. However, they frequently must be balanced when mining sensitive data [39].

Privacy-preserving ML can be deployed using MPC concepts, such as GC and oblivious transfers, and apply them to the construction of protocols that allow parties to train ML models without revealing individual party inputs.

For the implementation of this module, we analyze two state-of-the-art privacy-preserving ML protocols: *SecureML* and *ABY3*. 

#### 3.4.1. SecureML

*SecureML* [26] is a two-party efficient ML training protocol that enables the training of linear regression, logistic regression and neural network models. *SecureML* implements a server-aided multi-party computation in which the two parties that run the *SecureML* protocol are servers that receive data from a set of clients. At the end of the protocol, each client receives the model trained with the clients’ joint data. In *SecureML*, the two parties participating in the protocol must be non-colluding, meaning that they do not work together to defeat the protocol and retrieve the clients’ private inputs.

*SecureML* authors state that their solution is orders of magnitude faster than previous privacy-preserving techniques. To achieve these higher efficiency levels, *SecureML* splits the multiparty computation into two phases: the setup phase and the online phase. In the setup phase, each party tries to compute all the necessary multiplication triplet shares needed to perform the computations where the secret sharing of the multiplication triplets is achieved, using Yao GC and OT. After the setup phase, the online phase uses the multiplication triplets and the secret shared inputs to compute the required ML model.

For optimization, *SecureML* also deployed tests with homomorphic encryption to improve the intermediate results. The authors provide a complete overview in [26] and the theoretical proofs required to validate the functionalities. 

#### 3.4.2. ABY3

ABY3 [40] is a three-party general framework for privacy-preserving ML that trains linear, logistic, and neural network models, using three-party computation. ABY3 introduces several improvements that allow it to achieve greater performance than the *Secure ML* [26], in terms of the number of communications and the amount of data transferred, while allowing support for three or more parties (multiparty) computation instead of a two-party computation of *SecureML* [26]. ABY3 improvements include new fixed-point multiplication protocols for shared decimal values and a new general framework for efficiently converting between binary sharing (secret sharing of binary values), arithmetic sharing [41] and Yao’s sharing [42], with security against malicious adversaries.

From our analysis of the publicly available implementations of *SecureML* [43] and ABY3 [44], we found that ABY3 implementation is vulnerable to eavesdropping attacks. An eavesdropper could capture the random seeds that generate the randomness used to mask the secret inputs that are sent to other parties, which defeats the entire purpose of a multiparty computation protocol, enabling an external attacker to obtain the private inputs of one or multiple parties that participate on the ABY3 protocol. Due to that fact, we chose to use *SecureML* as our privacy-preserving ML protocol.

The data used to feed the *SecureML* protocol can be anonymous or private. In our architecture, anonymous data are never made public in plaintext form, which can prevent threats where an attacker uses background knowledge and other anonymous data to aggregate the information to find out if there are entities that belong to both sets of data. Our system never reveals anonymous data in cleartext, even in ML, so it is resilient to these types of attacks.

### 3.5. Cloud Middleware Module

The cloud middleware is solely responsible for storing and retrieving data from public and private storage. The implementation of the cloud middleware module is based on the jClouds framework, which enables us to deploy our solution to a vast number of supported cloud storage backends, including Amazon AWS, Google GCP, Microsoft Azure, and locally deployed OpenStack Swift instances [45]. Our implementation uses AWS, GCP, and Azure for our public cloud backends, each one receiving one of the anonymized file shards received from the encryption and indexing module, and our private cloud storage are based on the OpenStack Swift. This choice of storage backends allows us to work with both public and private cloud storage, using the same framework.

To improve the upload and download speed, we have made our process multithread. In the upload process, we split the fragments into 2 MB blocks. Then, ten threads are generated, where, for example, the first thread is responsible for uploading blocks 1, 11, 21, etc., storing the location where the block is stored in the cloud-of-clouds on the metadata server. By doing this, we always have ten blocks being loaded simultaneously, improving performance. For the download process, we generate ten threads and start the download for each block that was previously sent in such a way that, for example, the first thread is responsible for downloading blocks 1, 11, 21, etc. However, by doing this, the snippet may be received out of order as the modules that requested the download. To make the cloud middleware module, we order the blocks as they arrive, producing a continuous stream of data where all are ordered, reducing the need for other modules to handle the out-of-order reception of data.

## 4. Security Evaluation

In this section, we provide a threat analysis of the proposed system. We identify potential threats, how an attacker may attempt to exploit the system and the limitations we introduce to block that threat. 

### 4.1. External Sharing

An attacker can try to obtain sensitive data when sharing information with an external source. When a file is uploaded to the cloud, the owner loses control over their data. In a typical file storage scenario, there is not a risk score analysis regarding the shared information. To prevent an attacker from obtaining sensitive information from the users’ files, we decided to use K-Anonymity and RAPS to extract the sensitive data before sharing a file with the cloud, which gives the users the insurance that relevant private information is stored in a private cloud, not allowing the sharing of personally identifiable information with external sources.

Besides deleting the sensitive information, it is essential to mitigate the risk of extraction and retention of the dataset with extra fields by an attacker. We implemented an MPC module to ensure that no one party has access to the cleartext version of the data, which means that the knowledge extracted will be equivalent in both ends (only the output is revealed), and no extra information about the inputs is shared/known. 

### 4.2. Key Negotiation

The impersonation attack on the HTTPS protocol is due to the unauthorized access to the private key on the server [32], which can affect either current and previous communications. In order to mitigate this, we implemented OpenSSL inside SGX, providing an extra layer of security to the private key management. 

### 4.3. Public Cloud

Public clouds’ usage is motivated by price reductions and authentication mechanisms to protect user data, but the data are still in cleartext to the cloud provider, even if they encrypt it at rest. To mitigate this, we encrypted the data and stored it in multiple cloud providers, ensuring that even if a cloud provider obtains access to the encryption key, they will not access the entire content sent to the clouds by the user.

## 5. Performance Evaluation

In this section, we aim to evaluate the performance of our system. In Test 1, we started by testing the performance overheads that SGX might cause. In the second and third tests, we followed a different approach, as we focused on a dedicated machine and removed the performance overhead of the communication between the on-premise server and the Android device, which allows us to compare the related work without having to include the overhead/latency of the client/server communications. Thus, in the second test, we compared with the related work, and finally, in the third test, we addressed the system’s scalability.

### 5.1. Test 1—SGX Overhead

For this test, we defined three environments:

1. The on-premise server is used without SGX;
2. The SGX stores the private key and is only used on the public key operations of TLS;
3. Every cryptographic operation of TLS is handled on SGX, including a public key and symmetric key operations, such as the encryption and decryption of packets.

For this first test, we used a Xiaomi MI A2 lite (on-premise client) featuring Android 9, a Ubiquiti Uap-Ac-Pro Access Point (AP), and an Intel NUC6i7KYK for supporting our on-premise server. The smartphone was connected to the AP, using 802.11n AC with a standard 20 MHz channel width in the 5 GHz wireless frequency range. The network connectivity was a 1 GB upload and download internet.

For these runs and each test, we used five repetitions of each scenario.

Table 4 shows that there is an increase in the performance overheads in the usage of SGX. However, using SGX to protect the HTTPS certificate private key has less performance overhead, providing usable upload and download performance. When SGX is responsible for all cryptographic operations over HTTPS, the performance overhead is too much to have a usable system. Because of this, the on-premise server implementation only has SGX responsible for public-key cryptography operations that require access to the certificate’s private key. We also represent the standard deviation for the five repetitions of each sample.

### 5.2. Test 2—Comparison with Other Cloud-of-Clouds

For this test, we used Google GCP storage as our cloud storage, writing in different buckets. For the setup, we used a network powered by a NOS Power Router 4.0 router with a 200 Mb/s internet down-link and a 150 Mb/s internet up-link. The machine used to host the services and run the tests used by the evaluation is a machine running in an Intel i7-8750H 6-core CPU with a 512 GB SSD connected to the router via a gigabit Ethernet cable.

Having an SGX mode selected, we started by comparing our solution with other state-of-the-art offloading technologies to see our performance overhead of encryption, erasure coding, and the cloud-of-clouds offload.

In our testing, we used three files with sizes of 100 MB, 500 MB, and 1 GB, uploading and downloading them ten times for each file size. To reduce the possibility of having noisy readings caused by network latency, we placed the client and the on-premise server on the same machine.

Contrarily to the previous test, we tested with the cloud-of-clouds and with local swift instances on the same machine, allowing us to test the scenario without the overhead associated with the public cloud provider. For this, we used four instances of the OpenStack Swift storage container deployed on the same machine as the client and the on-premise server that simulates the off-premise public cloud storage that our solution uses.

We tested our solution against Charon, configured without pre-fetching and compression, and against *rClone*. We compare our solutions’ offload with the Charon, as it also uses multiple cloud providers for storing the information persistently. *rClone* tests should allow us to see the maximum throughput that the cloud backends allow, enabling us to verify how much offload performance is left on the table due to our overhead.

For sake of completion, we ran the same tests using Google GCP storage as our cloud storage backends to see how the network latency affected our results. Table 5 shows the results of our testing in seconds with an average of ten runs for each scenario. We also have the standard deviations to understand the min and max values of each run.

Regarding the tests with the cloud, we can achieve a better performance than Charon in the download process. However, in comparison with RClone, for 1 GB size files, we have a weaker performance. RClone only communicates with the cloud provider, so it has better performance. However, Charon has to wait for the cloud providers so that it can explain its results.

On the other hand, in the upload process, we only achieve the best results on the 1 GB file, as Charon seems optimized to small files because the difference between 100 MB and 500 MB is greater than 90 s.

Regarding the Swift tests, the download and upload times decrease for all solutions, as they are performed locally and there is no connection overhead with the cloud provider. RClone performs better in all the results, while our solution can achieve similar results as Charon, except for the 1 GB upload, where the performance of our solution is much better.

Swift tests also allow us to test the dependency of the public cloud providers, as RClone has better results than the other solutions. Compared with Charon, our solution has an overhead decrease of almost 50%.

### 5.3. Test 3—Scalability

In this test, we used the same setup provided in Test 2. We used a network powered by a NOS Power Router 4.0 router with a 200 Mb/s internet down-link and a 150 Mb/s internet up-link, and the machine used to host the services and run the tests used by the evaluation is a machine running in an Intel i7-8750H 6-core CPU with a 512 GB SSD connected to the router via a gigabit Ethernet cable.

In the architecture section, we stated that our architecture is built to be scalable. We achieved this by allowing multiple machines to run the same on-premise server code behind a load balancer. The load balancer allowed us to split the load equally among all the available machines running the on-premise server. In our implementation, we used *HAProxy* [46] as our load balancing technology with a round-robin configuration, meaning that the number of requests are divided equally among all the machines running the on-premise server. To verify whether our solution is indeed scalable, we devised a test where we have 1, 5, 10, and 20 concurrent users uploading files of 100 MB, 500 MB, and 1 GB, running ten times for each number of concurrent users and file size configuration. To simulate a more realistic use case, we used Google GCP as our storage backends. In our testing, we used three on-premise server instances running locally on the same machine where the requests were made. To help us compare our solution scalability with other state-of-the-art cloud offload technologies, we compared our solution with *rClone*. *rClone* is a standalone solution, meaning that it should have no scalability issues, as it connects directly with the cloud storage without any additional processing made locally. That *rClone* properties will help us to understand the maximum capacity that our testing environment has and to have a baseline on which to compare our solution. We did not use Charon on this test since it uses a caching service, making it only upload a file at a time instead of continuously uploading all the files, making it impractical for this performance comparison.

**Figure 3:**
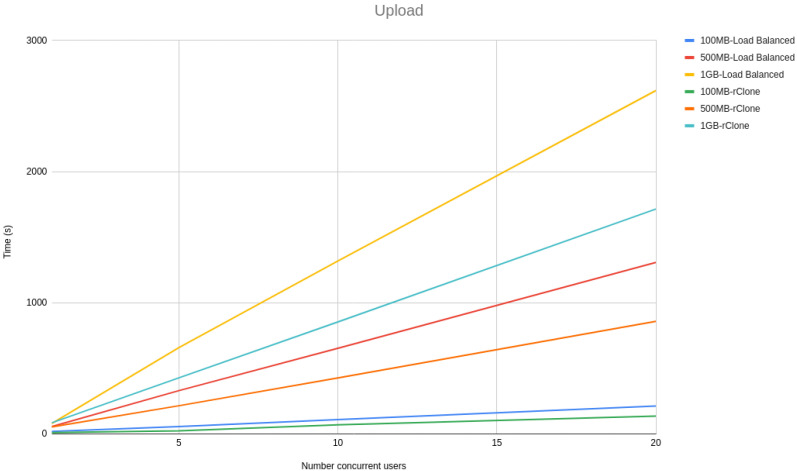
Upload scalability tests.

**Figure 4:**
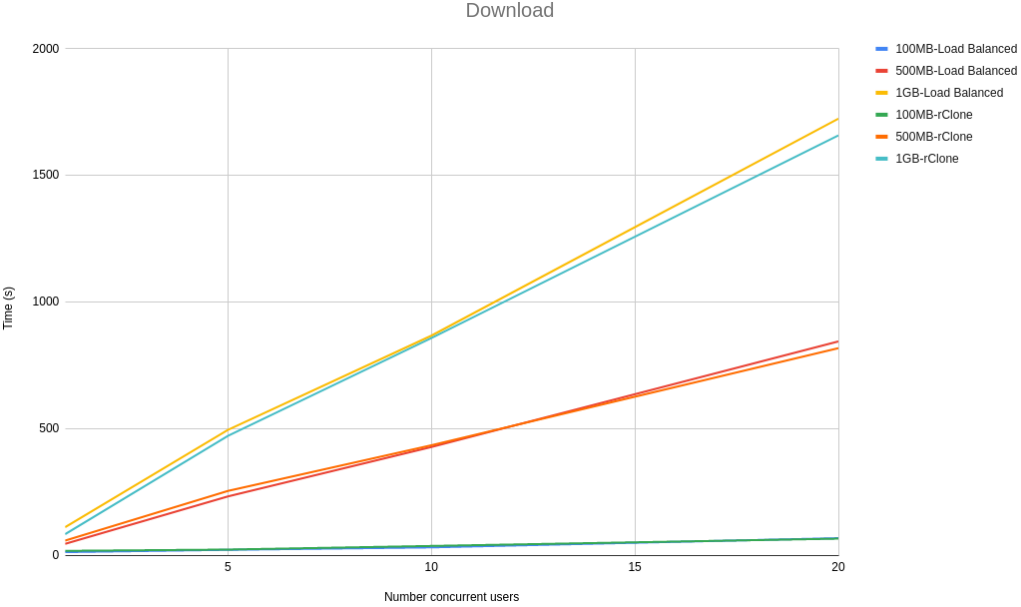
Download scalability tests.

Figure 3 and Figure 4 show a line graph of the meantime that took to, respectively, upload and download 100 MB, 500 MB and 1 GB files with 1, 5, 10 and 20 concurrent users.

From the line graphs, it is possible to see that the difference between our solution and *rClone* grows linearly with the size of the file and not with the number of users, meaning that our solution scalability is independent of the number of users but dependent on the file sizes. However, the growth is linear with the file size rather than being exponential, proving that our solution architecture is scalable.

## 6. Conclusions and Future Work

To the best of our knowledge, we described the first system that stores information in multiple cloud providers, focused on ensuring that users can share data and train ML models without exposing the data in cleartext, using MPC to compute the information jointly. The described system also uses known anonymization algorithms, such as K-Anonymity, storing the private information on the private cloud and storing the anonymized data in cloud-of-clouds storage.

With the evaluation we made of our data offload system, we demonstrated that our solution is scalable and can benefit the user experience, compared to the related work. In this scenario, the major disadvantage is using SGX to perform all calculations and manage encryption and decryption processes, as the performance overhead limits the system. Contrarily, if we manage only the private key inside the enclave, there is no overhead associated with this usage, ensuring that the private key never leaves the device.

This solution helps to achieve secure storage of information at a reduced price of the cloud providers, maintaining the data encrypted outside of the perimeter of the infrastructure to allow sharing with other entities to maintain user privacy. In addition, it can help to solve some open research challenges, such as designing secure privacy protection schemes to ensure that sensitive information in shared data is hidden, and designing efficient and secure third-party privacy protection schemes to support more machine learning algorithms [1,5].

There are still some challenges to address in the current implementations. The most important is related to the future developments of the multiparty computation and the scalability issue associated with such computations, but principally in the auditing and verification of the implementations.

As future work, we can also enhance the identification and removal of the identifiers, and redacting can be a possibility of using a PDF or other type of document to remove or create blocks that do not allow other parties to read from the file [47]. A user can select confidential information with a mark redaction tool. Then, the information marked as redacted will be encrypted and restricted to authorized users. 

## Figures and Tables

**Table 1:** Advantages and disadvantages of standalone cloud storage.

Advantages	Disadvantages
The user controls the location and encryption process of each file	If the user encrypted the files and lost the encryption keys, it loses access to data
	Sharing encrypted information with other users is impossible
	Performance limitations–the encryption process can be expensive

**Table 2:** Advantages and disadvantages of hybrid cloud storage.

Advantages	Disadvantages
Encryption in the *hybrid* component (in a trusted entity)	Single point of failure
Share information with other users	
Allows caching services	

**Table 3:** Advantages and disadvantages of distributed cloud storage.

Advantages	Disadvantages
Maintains control of the files	Performance limitations, the encryption process is done locally
Share information with other users	
Allows caching services	

**Table 4:** SGX overhead testing in MB/s.

	(1) On-premise Server without SGX		
File Size	100 MB	500 MB	1 GB
Upload MB/s	12.45 ± 0.43	11.90 ± 0.63	12.04 ± 0.84
Download MB/s	14.10 ± 2.76	13.66 ± 1.33	14.00 ± 1.44
	(2) On-premise Server SGX only PK		
File Size	100 MB	500 MB	1 GB
Upload MB/s	9.23 ± 0.33	10.90 ± 0.43	10.49 ± 0.14
Download MB/s	10.10 ± 1.05	12.45 ± 2.48	14.12 ± 1.85
	(3) On-premise Server SGX PK + SK		
File Size	100 MB	500 MB	1 GB
Upload MB/s	1.88 ± 0.02	1.54 ± 0.43	1.54 ± 0.33
Download MB/s	1.35 ± 0.47	1.37 ± 0.48	1.38 ± 0.08

**Table 5:** Off-premise data offload performance when compared with Charon and rClone, in seconds.

	DOWNLOAD		
CLOUD	100 MB	500 MB	1 GB
Our Solution (s)	13.360 ± 1.427	45.718 ± 18.871	112.048 ± 2.269
Charon (s)	14.884 ± 0.752	77.998 ± 3.245	209.172 ± 7.658
Rclone (s)	18.486 ± 1.794	58.727 ± 5.022	84.364 ± 33.673
	**UPLOAD**		
**CLOUD**	**100 MB**	**500 MB**	**1 GB**
Our Solution (s)	18.657 ± 2.335	56.079 ± 0.451	79.031 ± 3.244
Charon (s)	8.904 ± 0.117	102.610 ± 0.781	152.324 ± 2.645
Rclone (s)	9.623 ± 0.524	52.657 ± 8.541	84.473 ± 5.368
	**DOWNLOAD**		
**SWIFT**	**100 MB**	**500 MB**	**1 GB**
Our Solution (s)	2.668 ± 0.236	9.870 ± 0.416	25.237 ± 3.365
Charon (s)	2.222 ± 0.117	10.333 ± 0.263	22.924 ± 0.293
Rclone (s)	1.230 ± 0.037	10.333 ± 0.437	12.838 ± 0.580
	**UPLOAD**		
**SWIFT**	**100 MB**	**500 MB**	**1 GB**
Our Solution (s)	5.537 ± 0.770	15.601 ± 2.162	34.354 ± 5.615
Charon (s)	8.904 ± 0.438	31.692 ± 0.800	65.567 ± 1.906
Rclone (s)	0.933 ± 0.150	6.126 ± 0.601	12.079 ± 0.426
